# A database of multimodal myography and hand kinematics during realistic daily life activities

**DOI:** 10.1038/s41597-025-05852-6

**Published:** 2025-08-28

**Authors:** Daniel Andreas, Dominik Werner, Zhongshi Hou, Anany Dwivedi, Claudio Castellini, Philipp Beckerle

**Affiliations:** 1 Chair of Autonomous Systems and Mechatronics, Friedrich-Alexander-Universität Erlangen-Nürnberg, 91054 Erlangen, Germany; 2 Artificial Intelligence (AI) Institute, Division of Health, Engineering, Computing and Science, University of Waikato, Hamilton, 3216 New Zealand; 3 Assistive Intelligent Robotics Lab, Friedrich-Alexander-Universität Erlangen-Nürnberg, 91054 Erlangen, Germany; 4 Department of Artificial Intelligence in Biomedical Engineering, Friedrich-Alexander-Universität Erlangen-Nürnberg, 91054 Erlangen, Germany

**Keywords:** Rehabilitation, Biomedical engineering

## Abstract

This paper introduces MyoKi, a database capturing multimodal myography and hand kinematics during various realistic daily life activities. MyoKi emphasizes the complexity of real-world settings, addressing limitations of existing databases, which often reflect controlled laboratory conditions. The database includes two subsets of participants designed to evaluate different sensor configurations. Both subsets contain surface electromyography (sEMG) and inertial measurement unit (IMU) data, along with hand kinematics covering 18 finger and wrist joints. For the second subset, additional force myography (FMG) data was collected. The database captures hand movements of 35 participants performing 74 tasks, with varying arm orientations and movements involving different grips and motions. By offering detailed participant profiles and systematically categorizing each task, the MyoKi database enables in-depth exploration of task complexity, sensor influence, and the impact of demographic and anthropometric factors on control system performance. The database is designed to facilitate research in continuous hand control, enhancing the robustness and reliability of myoelectric devices for daily activities, moving towards user-friendly and effective control of robotic and prosthetic hands.

## Background & Summary

Recent advancements in pattern recognition have enabled gesture-based control of both virtual and real robotic hands, as well as myoelectric hand prostheses^1–5^. The classification of various movement patterns facilitates the execution of fundamental daily tasks and has already been applied to commercially available myoelectric prosthetic hands^6^. However, this form of control cannot fully restore the hand’s functionality, as users are limited to a predefined set of movement patterns. The reliance on predefined movement patterns makes it challenging to perform more complex tasks requiring fine, individual, and continuous finger movements that vary in dynamics.

This control limitation is one of the primary reasons for the historically low acceptance of myoelectric prostheses among individuals with hand or forearm amputations^7^. Consequently, recent research has focused on enabling continuous control of artificial hands. Numerous databases containing electromyography (EMG) data and hand kinematics have been collected to drive progress. These databases can be used to facilitate machine learning-based decoding of muscular activity to control robotic or virtual hands and can provide valuable insight to improve prosthetic hand control. Some of these databases are compiled within the Ninapro database^8–12^, while others are found in works by Furmanek *et al*.^13^, Hu *et al*.^14^, Jiang *et al*.^15^, Malešević *et al*.^16,17^, Matran-Fernandez *et al*.^18^, and Turgunov *et al*.^19^. While these databases encompass a wide range of individuals and movement patterns, their relevance for practical applications is limited, as they primarily reflect controlled laboratory conditions rather than realistic everyday scenarios. To ensure high signal quality, movement patterns were typically performed from a specific resting position in those databases, with regular breaks to prevent muscle fatigue and the associated signal degradation.

To make realistic statements about the ability to decode muscular activity into hand motions, it is crucial to account for the complexity of daily life. Thus, our focus was to define complex tasks that require different arm orientations and allow the user to fulfill the task in their preferred way. Although tasks and required grips were predetermined, the precise movement sequences were not strictly defined. Participants performed tasks that involved not only hand movements but also broader body motions (e.g., reach-to-grasp tasks with varying vertical and horizontal distances), helping to identify potential interferences and enhance the robustness of control systems for real-world applications.

The MyoKi database presented in this work consists of two subsets of participants sharing the same data acquisition protocol. Subset 1 contains data from 25 participants (P01 to P25), and subset 2 contains data from another 10 participants (P26 to P35). In both subsets, surface electromyography (sEMG) sensors were used, of which many contain a 3-axis gyroscope and a 3-axis accelerometer. The sensors were distributed across the participant’s right arm. For the second subset of participants, additional FMG data were acquired along with EMG. By incorporating FMG, we aim to mitigate the high noise levels associated with EMG sensors^20–22^, thereby potentially improving the robustness of robotic hand control. Previous studies have already shown an increase in hand classification using a combination of EMG and FMG data compared to each data individually^20,22–26^. With the MyoKi database, we want to provide the tools to explore whether those benefits also transfer to regression tasks. Although FMG has been used successfully to record muscle activity in the past, we are not aware of any existing databases that incorporate FMG data along with EMG and hand kinematics data.

This database aims to enable testing the combination of various sensor modalities to maximize the decoding accuracy of hand motions during daily life. Further, the database allows to investigate which tasks or finger joints are more difficult to decode. The results are expected to offer insights into optimizing data collection regarding sensor selection/location and task design. By acquiring demographic and anthropometric information from the participants, researchers can study the influences of age, sex, and body fat on the ability to decode the acquired biosignals into wrist and hand motions to improve robotic hand control by personalized control algorithms. Ultimately, the goal is to provide a realistic database that is close to daily-life situations, allowing for a better translation from offline to online experiments. This shall make the artificial hand control more robust and move the control closer to the real hand.

## Methods

The data collection from 35 participants with no upper limb mobility limitations within two subsets was in accordance with the Institutional Ethics Committee of the Friedrich-Alexander-Universität Erlangen-Nürnberg (24-439-S 2024-12-11). Of the original 38 participants, 3 were excluded due to corrupted task labels. The participants were selected to cover various age groups (18-29: 11 participants, 30-44: 8 participants, 45-59: 9 participants, 60+: 7 participants) and to obtain an even distribution between sex (17 female, 18 male). After explaining the experiment and obtaining informed consent for participation and data sharing, participants’ weight (76.6 ± 14.7 kg) and forearm circumference (27.1 ± 2.7 cm) were measured. Additionally, participants’ height (174.4 ± 8.6 cm), handedness (right: 31, left: 4), as well as their geographic and ethnic backgrounds, were recorded to help interpret differences in the ability to decode muscular activity into continuous finger and wrist motions.

Since body fat can influence the quality of signals from muscular activity^27,28^, a skinfold caliper was used to measure subcutaneous fat thickness at the triceps. This site, among others, is commonly utilized in body fat assessment using established methods such as the ones by Durnin & Wormersley^29^ or Jackson & Pollock^30^. Because data is collected exclusively from the participant’s right arm, local subcutaneous fat is more relevant than total body fat percentage. Therefore, a single-site skinfold measurement at the triceps was considered most suitable. The measured skinfold thickness at the triceps across participants was 17.6 ± 8.7 mm. See the chapter “Data Records” on how to access the table containing all participant information.

### Sensor Layout

**Fig. 1:**
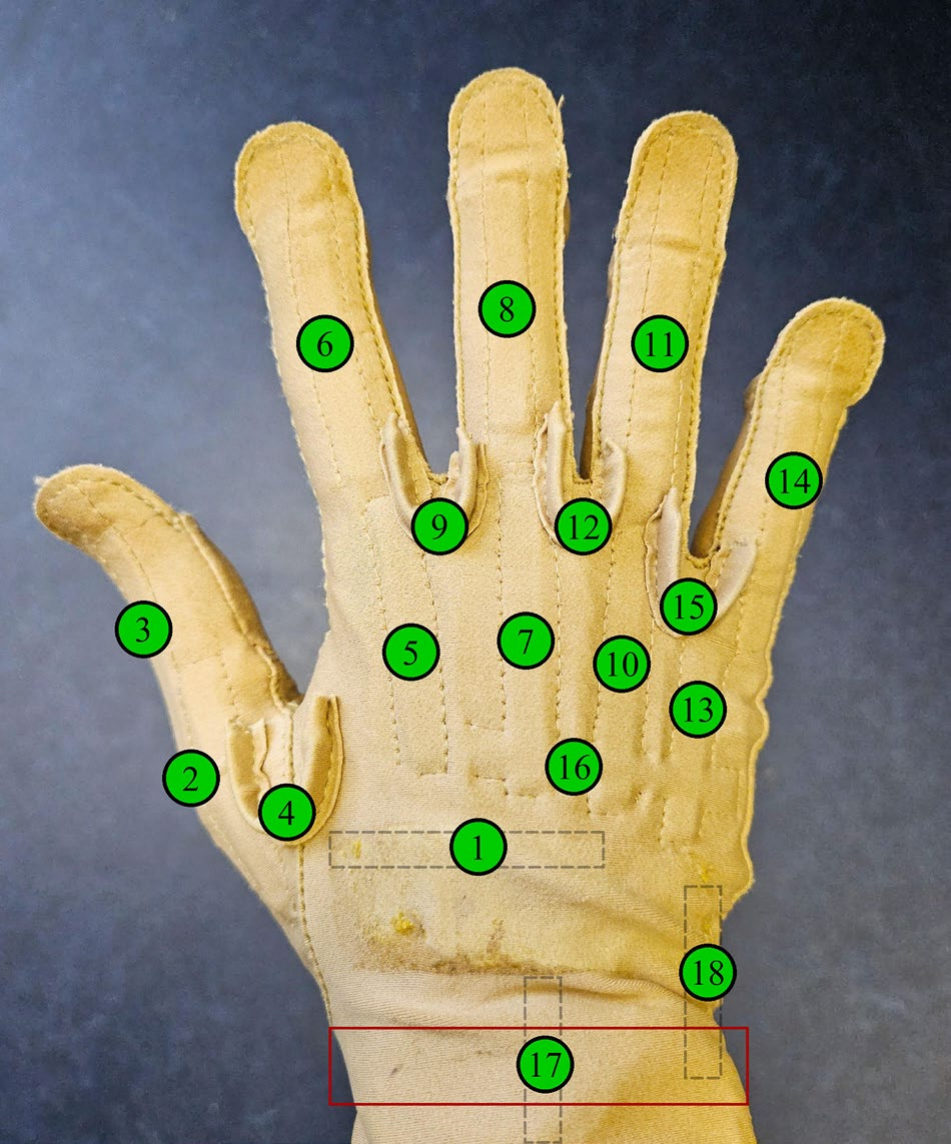
Sensor layout of the CyberGlove by CyberGlove Systems LLC, CA, USA, to acquire data from 18 hand and wrist joints. The location of the strap to improve the signal quality of wrist sensors 17 and 18^31^ is marked in red.

Before starting the data acquisition, all sensors were attached to the participant. To measure hand kinematics in form of joint flexion, the CyberGlove by CyberGlove Systems LLC, CA, USA, was used. It acquires data from 18 hand and wrist joints using flex sensors as shown in Fig. 1. A strap was attached around the wrist, which according to Jarque-Bou *et al*.^31^ improves the signal quality of the wrist sensors 17 and 18 (see Fig. 1). We followed the calibration procedure by Belić *et al*.^32^ to convert the arbitrary flexion values from channels 1 to 15 (see Fig. 1) into joint angles. We collected the calibration data from one female and one male participant who matched the average hand size to convert the kinematic data into angles for all female and male participants in the database, respectively. Recent anthropometric hand data (length and breadth) from Czech Republic^33^ and Spain^34^, which are both geographically close to Germany where the data acquisition took place, were averaged for females (length: 180 mm, breadth: 77 mm) and males (length: 195 mm, breadth: 87 mm). This roughly matches the median values for the German population in the year 2000, with median values for 20 to 24 years old female (length: 178 mm, breadth: 76 mm) and male (length: 194 mm, breadth: 87 mm) participants^35^. Assuming linear behavior of the CyberGlove’s flex sensors, a gain factor and offset value can be calculated by obtaining two values per sensor at specific angles (typically 0^°^ and 90^°^, for a complete guide see the work by Belić *et al*.^32^). The calibrated joint angles were then calculated by applying a gain and offset value to the sensor readings as in the following equation:

1 $$\begin{array}{rcl}angle & = & gain\times (sensor\_value\,-\,offset),\,with\\ gain & = & \frac{angl{e}_{1}\,-\,angl{e}_{2}}{sensor\,\_valu{e}_{1}\,-\,sensor\,\_valu{e}_{2}},\,and\\ offset & = & sensor\_valu{e}_{1}\,-\,\frac{angl{e}_{1}}{gain}.\end{array}$$

*sensor*_*value* denotes the current sensor value to be converted into a joint angle. *angle*_1_ and *angle*_2_ are the calibration angles with *sensor*_*value*_1_ and *sensor*_*value*_2_ as the corresponding sensor readings by the CyberGlove for those angles. To calibrate channel 16 (palmar arch), the method by Gracia-Ibáñez *et al*.^36^ was applied. Therefore, the palmar arch was measured at a neutral position (0^°^), and another sensor value was obtained at maximum palmar arch flexion. The angle at maximum flexion was measured using a goniometer between the line connecting the index and middle knuckle with the line connecting the ring and little knuckle. Equation (1) was then again used to convert the sensor values into joint angles.

The wrist sensors 17 and 18 were individually calibrated for each participant using data from the CyberGlove acquired during the experiment, as across-subject generalization, applied to all other sensors, did not yield satisfactory results. Among other tasks, participants performed radial/ulnar deviation (task 73) and wrist flexion/extension (task 74) during data acquisition. The minimum and maximum values observed during these tasks were extracted and averaged across all repetitions. These values were then mapped to the angles provided by Nizamis *et al*.^37^, measured across 20 participants using a goniometer: maximum wrist flexion (+79^°^), extension (−63^°^), radial deviation (−19^°^), and ulnar deviation (+35^°^). Equation (1) was then applied again to calibrate the values from sensors 17 and 18 accordingly. It should be noted that the calibrated hand kinematics provided as joint angles should be used carefully, since the joint angles could only be approximated from the raw flexion values.

**Fig. 2:**
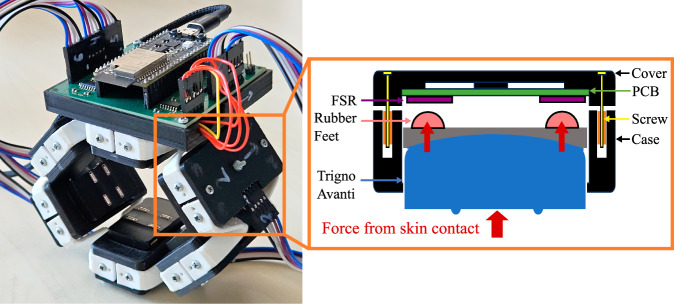
Picture showing the multimodal bracelet by Andreas *et al*.^38^ that was used to acquire EMG (in both subsets) and FMG data (in the second subset). The bracelet spaces EMG channels 1 to 6 equally around the forearm. For the first subset, a dummy PCB was used to ensure consistent EMG sensor placement, maintain comparable protrusion of the EMG sensor, and guarantee reliable skin contact across both subsets. The right shows a detailed view of a module, which combines a Trigno Avanti sensor with 4 FSR-based FMG channels at a single location.

**Fig. 3:**
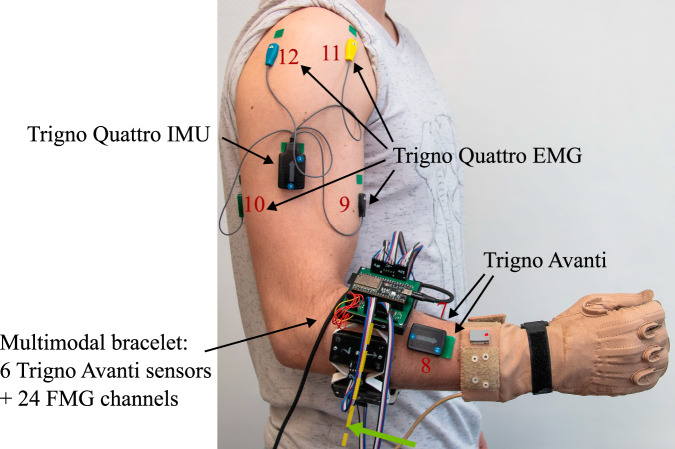
Sensor layout on participant showing the location of all EMG sensors and the multimodal bracelet by Andreas *et al*.^38^ containing Trigno Avanti EMG sensors 1 to 6 and 24 FMG channels. All Trigno Avanti sensors are oriented with the arrows pointing in the distal direction, while the Trigno Quattro sensor is pointing in the proximal direction. This defines the axes orientation of the gyroscopic and accelerometer data. This layout was used for both subsets of participants, with the only difference that no FMG data was recorded for the first subset.

To acquire muscular activity, eight Trigno Avanti and one Trigno Quattro EMG sensor by Delsys Incorporated, Natick, USA, were used in mode 65 (see SDK) to acquire EMG signals at 1259 Hz, which were then internally upsampled to 2000 Hz according to the datasheet. While the Trigno Quattro sensor contains four separately attachable EMG channels and a single inertial measurement unit (IMU) with a 3-axis gyroscope and 3-axis accelerometer (measuring range up to 16 g) in the main unit, the Trigno Avanti sensors each contain a single EMG channel and IMU. The IMU of the Trigno Avanti and Trigno Quattro sensors both acquire signals at 148 Hz according to the datasheet. As shown in Fig. 3, the sensor layout comprised a total of 12 EMG channels and nine IMUs for data acquisition, which were located across the participant’s right arm. We used the bracelet by Andreas *et al*.^38^ without the additional FMG sensors for the first subset of the database to equally space EMG sensors 1 to 6 around the participant’s forearm to ensure consistent placement and optimal use of the available surface area. This approach establishes a reproducible relationship between each sensor channel and the underlying muscles, while also reflecting the practical constraints often encountered in prosthetic sockets, where targeting specific muscles is typically not feasible. Moreover, muscle-specific targeting is particularly challenging in individuals with higher subcutaneous fat levels and may introduce placement errors. For the second subset of the MyoKi database, the multimodal bracelet by Andreas *et al*.^38^ was used with the additional FMG sensors (see Fig. 2) to combine EMG channels 1 to 6 with 24 force-sensitive resistor (FSR)-based FMG channels (4 FMG channels per module). The individual modules of the multimodal bracelet were designed to allow very small vertical movements (<1 mm) of the Trigno Avanti modules to transfer forces to the FSRs of type 400 Short by Interlink Electronics Inc., Camarillo, CA, USA, which are placed superficial to the EMG sensors, allowing EMG, IMU, and FMG readings at the same muscle location. For both participant subsets, we ensured consistent bracelet placement and standardized the protrusion of the EMG sensors from the modules to maintain reliable skin contact. See the work by Andreas *et al*.^38^ for further information and functional tests of the multimodal bracelet. Additional information on signal range, unit, and frequency can be found in the section “Data Records”.

**Fig. 4:**
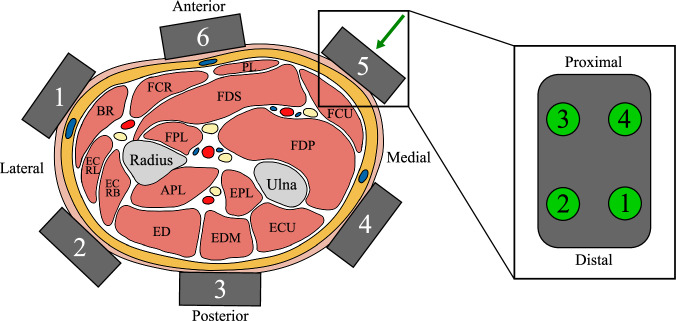
Sensor layout of the EMG/multimodal bracelet on the right forearm. The direction of the view can be depicted by the green arrow in Fig. 3 (distal to proximal). The gray rectangles represent the six modules of both the EMG bracelet and the multimodal bracelet. The layout of the FMG sensors is shown in the detailed view on the right (top view). Muscle abbreviations (left to right): Brachioradialis (BR), Extensor carpi radialis longus (ECRL), Extensor carpi radialis brevis (ECRB), Flexor carpi radialis (FCR), Flexor pollicis longus (FPL), Abductor pollicis longus (APL), Extensor digitorum (ED), Palmaris longus (PL), Flexor digitorum superficialis (FDS), Extensor pollicis longus (EPL), Extensor digiti minimi (EDM), Flexor carpi ulnaris (FCU), Flexor digitorum profundus (FDP), Extensor carpi ulnaris (ECU).

The skin for each sensor location was cleaned with alcohol wipes before application. The following protocol was followed to ensure consistent sensor positioning across participants:

• EMG/Multimodal bracelet^38^: Bracelet orientation with the arrows of the Trigno Avanti sensors pointing toward the participant’s hand. The bracelet was located at the largest circumference of the forearm, which shall prevent the bracelet from slipping during task execution and ensure good contact between the skin and electrodes. Module 6 of each bracelet was then positioned to align with the participant’s right arm’s cubital fossa while the participant was standing as in Fig. 3. Figure 4 illustrates the arrangement of sensors within the EMG bracelet and the multimodal bracelet. It shows a cross-section of a right forearm from the distal to proximal direction, with the approximate locations of the six modules positioned above the underlying muscles^38^. The figure also defines the consistent order of FMG channels for each module that was used for the second subset of the MyoKi database.

• EMG 7 & 8: On the right forearm between the CyberGlove (see Fig. 1) and the EMG/multimodal bracelet in distal direction. EMG 7 is located between modules 5 and 6 to target the flexor digitorum superficialis, while EMG 8 is located between modules 1 and 2 of the EMG/multimodal bracelet (see Fig. 4).

• EMG 9 & 10: Center of the right biceps brachii and triceps brachii, respectively.

• EMG 11 & 12: Intersection between the right lateral deltoid and the front/rear deltoid, respectively.

• Trigno Quattro IMU: Below the right deltoid, in between biceps brachii and triceps brachii.

### Experimental Setup

**Fig. 5:**
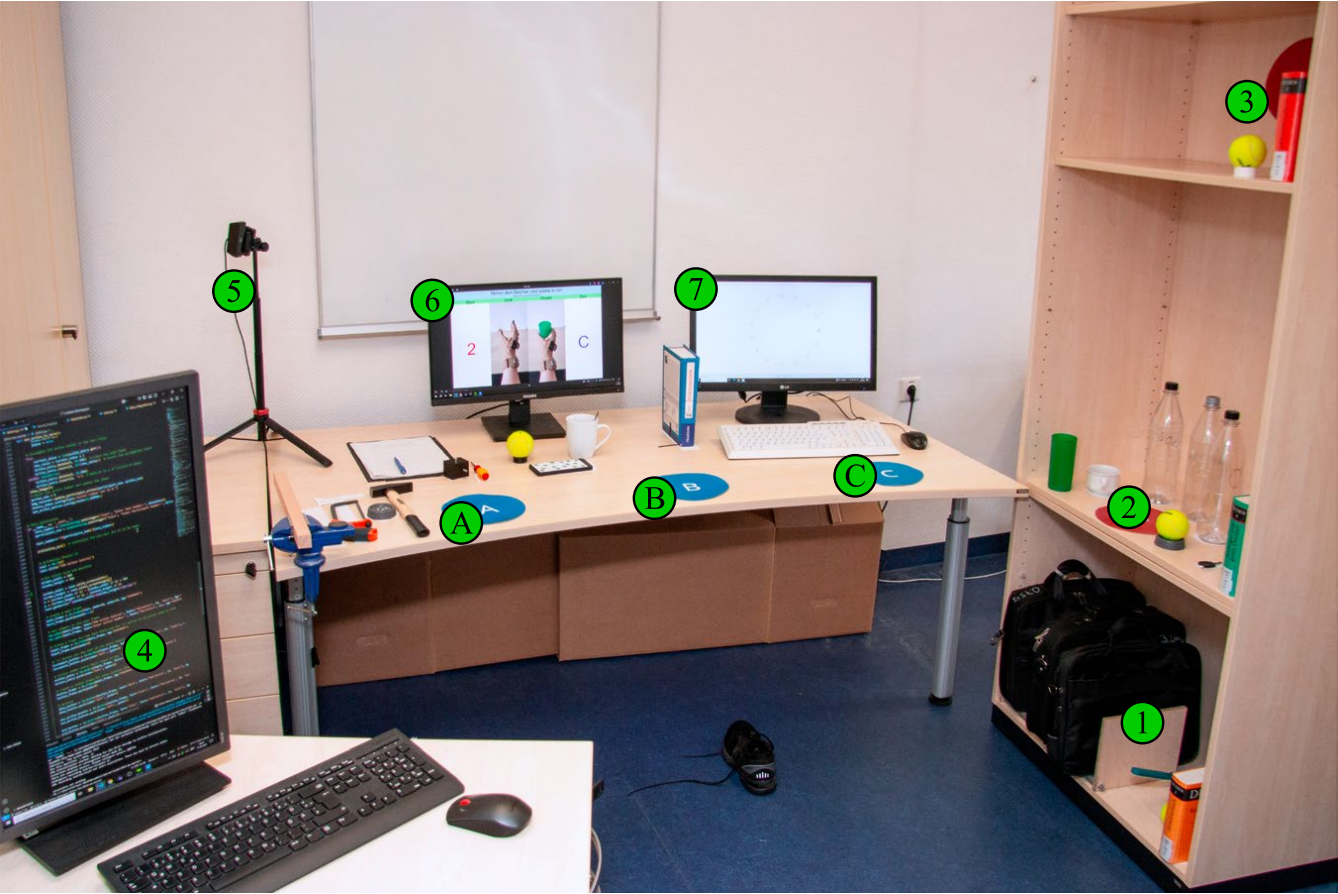
Experimental setup for the data acquisition. Markers 1, 2, and 3 show the object locations for the pick-and-place tasks, while A, B, and C mark the target locations. The experimenter (4) manually labeled the data with task ID and repetition. The participant’s actions were recorded on video by a webcam (5) to correctly annotate the data in case of errors by the experimenter or participants. The task instructions were displayed on a screen (6). A second screen (7) was provided to perform the clicking and typing tasks.

After the sensor application and a brief instruction, the participants were asked to perform a series of predefined daily tasks displayed on a screen as in Fig. 5 (marker 6), which shows the experimental setup. The markers 1, 2, and 3 in Fig. 5 mark the object locations for the pick and place tasks, while markers A, B, and C mark the target locations to place the objects. The respective distances between object location 2 and target locations A, B, and C are 1.5 m, 1.0 m, and 0.5 m, respectively. During the experiment, participants were knowingly recorded on video by a webcam (marker 5). The video data was only used for accurate annotation of the acquired data and is not published.

The experimenter (at marker 4 in Fig. 5) set the labels, such as task ID and repetition, manually in a computer program and signaled the participant the start of each task. The participants were instructed to place their hand near the object before the start of each task, ready to grasp the object or perform the upcoming task. This allowed precise labeling of the data and prevented the inclusion of unwanted movements that are not associated with the respective task. The transition phases in between tasks were labeled with zero, while tasks were labeled with their respective task ID. Participants performed a sequence of 74 different tasks in a fixed order. After completing the full set of tasks, the process was repeated 6 times to achieve a roughly even distribution of muscle fatigue across tasks and create a more realistic setting, which caused participants to include slight variations in task execution since repetitions were not performed back to back.

The experimental setup was inspired by the work of Nowak *et al*.^39^, who created a setup to train and assess the performance of myoelectric hand prosthesis control. The setup focuses on repeatability and postural variations during task execution to mimic complex daily-life situations. We extended the setup with more complex tasks to emphasize fine individual finger movements. The selected tasks vary regarding their location (below table height, at table height, above table height, sitting), motion distance (none, short: ≤0.75 m, medium:  > 0.75 m ∧ < 1.25 m, long: ≥1.25 m) and direction (none, lateral, medial), grasp (cylindrical, spherical, hook, tripod, pinch, lumbrical, complex), wrist action (none, supination/pronation, deviation, flexion, complex), required force (low, medium, high), and whether the execution requires unimanual or bimanual actions. Since all sensors were attached to the participant’s right arm, all unimanual tasks, such as pick and place tasks, were performed using the right hand. Table 1 shows three of the 74 tasks as an example, which were categorized with respect to the aforementioned variations (see the Chapter “Data Records” on how to access the complete table) and are described in the following:

- Task 24 - Take the key from 2 and place it on B: This task is performed at table height using a tripod grasp covering a medium distance of 1.0 m in medial direction. No wrist action is required for this pick-and-place task. Due to the low weight of the object, the task requires only low force and is performed unimanually.
- Task 45 - Take the 3 kg laptop bag from A and place it on 1: This task directly follows the previous task 44 to place the 3 kg laptop bag from 1 to A. Due to the bag’s position, this task is categorized to be performed below table height. The object is moved a long distance of more than 1.5 m in lateral direction using the hook grip. This task requires no wrist action and is performed unimanually, but requires a high force due to the increased 3 kg weight of the bag.
- Task 58 - Tie the laces of the shoe and untie them again: This task is executed while sitting. It does not involve the re-placing of an object. The task requires bimanual complex finger and wrist motions at a low force.

**Table 1:** Showing task 24, 45, and 58 from left to right with their corresponding categorization as an example.

		Take the key from 2 and place it on B.	Lift the 3 kg laptop bag from A and place it on 1.	Tie the laces of the shoe and untie them again.
Object/Task location (vertical)	Below table height		•	
	At table height	•		
	Above table height			
	Sitting			•
Horizontal distance	None			•
	Short			
	Medium	•		
	Long		•	
Motion direction	None			•
	Medial	•		
	Lateral		•	
Grasp	Cylindrical			
	Spherical			
	Hook		•	
	Tripod	•		
	Pinch			
	Lumbrical			
	Complex			•
Wrist action	None	•	•	
	Supination/Pronation			
	Deviation			
	Flexion			
	Complex			•
Force	Low		•	•
	Medium			
	High		•	
Manual actions	Unimanual	•	•	
	Bimanual			•

Note that this table is transposed compared to the complete task table with all 74 tasks in the online repository.

The tasks were designed to eliminate the need for object resets between repetitions, streamlining the data acquisition process and maximizing the amount of data collected within a given time. For example, after task 24, where a key is moved from position 2 to B, task 25 requires returning the key from B to 2, effectively resetting the object to its original position. Additionally, the motion direction of task 25 shifts from medial to lateral. To assess the impact of movement distance on muscular activity and thus decoding performance, the pick-and-place task with the key was additionally performed over both a short distance (0.5 m from 2 to C) and a long distance (1.5 m from 2 to A). Other pick-and-place tasks involved various objects requiring different grasp types, as well as variations in vertical placement (e.g., locations 1 and 3) and object weight. For instance, task 45 was performed using both a medium-weight bag (1.5 kg) and a lighter bag (0.75 kg) to evaluate the impact of variations in required force to complete the task.

In addition to these tasks, more complex movements emphasizing fine individual finger control were included. These involved common activities such as tying and untying shoelaces (task 58), opening and closing a water bottle (task 7 & 8), opening a book and turning pages (task 57), and handwriting a sentence on paper (task 61). Contemporary interactions, such as clicking targets on the PC (task 62), typing on a keyboard (task 63), and tapping targets on a smartphone using the thumb (task 59), were also incorporated. Additional tasks focused on wrist movements, such as using a duster (task 46–48) or screwing and unscrewing a horizontal screw (task 54 & 55). Lastly, tasks were included to capture the full range of motion for each finger, involving five repetitions of flexion and extension (task 65–69), fist clenching and stretching (task 70), as well as adduction and abduction (task 71). Wrist movements such as supination/pronation, radial/ulnar deviation, and flexion/extension (task 72–74) were also included.

### Data Processing

After data acquisition, the IMU, FMG (in the second subset), and hand kinematics data were upsampled to match the 2000 Hz sampling rate of the EMG signal. All signal modalities were collected by a single host PC, with each incoming sample labeled with an absolute timestamp from a common time server to ensure synchronization across the database. To temporally align signals sampled at different frequencies, we used nearest-neighbor alignment based on timestamp proximity. Specifically, we applied a non-interpolative method that assigns, for each timestamp in the higher-frequency signal, the most recent preceding (or equal) value from the lower-frequency signal. This approach is equivalent to a zero-order hold (ZOH) strategy, wherein the last known sample is assumed constant until a new sample becomes available. This preserves the original sample values without introducing interpolation artifacts. All signals were then compiled into a single MATLAB data file (*.mat) for each participant.

## Data Records

The collected data and all supplementary files, such as the task list and participant information, are available on figshare^40^ (10.6084/m9.figshare.28696778):

- P01.mat ... P35.mat: Matlab data files containing all sensor data during task execution for each participant.
- Task_categorization.xlsx: Microsoft Excel file that contains a table with all tasks and their corresponding Task ID. The tasks are categorized as in Table 1.
- Participant_information.xlsx: Microsoft Excel file that contains all relevant participant information such as participant ID (P01 ... P35) and corresponding age, sex, height, weight, forearm circumference, handedness, skinfold measurement at the triceps, as well as their geographic and ethnic backgrounds. The file further contains notes on anomalies during data acquisition, such as missing repetitions or loose sensors.
- NinaproDB7_replica.mat: Matlab data file containing sensor data by one participant replicating the acquisition protocol from Ninapro database 7^11^. It can be used for direct comparisons with the original NinaproDB7 to validate signal quality.
- Glove_calibration.xlsx: Microsoft Excel file that contains calibration data of the CyberGlove collected from one female and one male participant with average hand sizes.

The Matlab data files containing sensor data for each participant (P01.mat ... P35.mat) are described in the following. Note that FMG data is only available in the second subset of the MyoKi database (P26 to P35).

- task: Task labels for each sample according to the file “Task_categorization.xlsx”. Label “0” marks the transition phase between tasks. Data shape: *number of samples × 1*.
- grasp: Task labels translated into grasps (0: Rest/Transition, 1: Cylindrical, 2: Spherical, 3: Hook, 4: Tripod, 5: Pinch, 6: Lumbrical, 7: Complex) using the information from “Task_categorization.xlsx”. Data shape: *number of samples* × *1*.
- repetition: Repetition label for each sample, ranging from 1 to 6. Data shape: *number of samples* × *1*.
- frequency: Sampling frequency of all signals. Data shape: *1* × *1*.
- timestamp: Relative timestamp in seconds for each sample starting with 0.0 s. Data shape: *number of samples* × *1*.
- emg: EMG data from eight Trigno Avanti sensors and one Trigno Quattro sensor acquired in Volts at 1259 Hz and internally upsampled to per-second mean frequencies of 1998.96 ± 3.93 Hz across participants. The average within-participant standard deviation of the sampling frequency was 41.50 Hz. Data shape: *number of samples* × *12 (number of EMG channels)*. The order of the EMG channels matches the one from Figs. 3 and 4.
- acc: 3-axis accelerometer data from eight Trigno Avanti sensors and one Trigno Quattro sensor acquired in multiples of the earth’s gravitation, denoted by g at per-second mean frequencies of 146.91 ± 0.05 Hz across participants. The average within-participant standard deviation of the sampling frequency was 5.60 Hz. Data shape: *number of samples)* × *27 (number of IMUs* × *3*. The order of the ACC channels matches the one from Figs. 3 and 4 and follows the scheme of the EMG channels: acc_1_x, acc_1_y, acc_1_z, acc_2_x, ...
- gyro: 3-axis gyroscopic data from eight Trigno Avanti sensors and one Trigno Quattro sensor acquired in degrees per second (dps) at per-second mean frequencies of 146.91 ± 0.05 Hz across participants. The average within-participant standard deviation of the sampling frequency was 5.60 Hz. Data shape: *number of samples* × *27 (number of IMUs* × *3*). The order of the ACC channels matches the one from Figs. 3 and 4 and follows the scheme of the EMG channels: gyro_1_x, gyro_1_y, gyro_1_z, gyro_2_x, ...
- glove: Flexion from 18 hand and wrist joints from CyberGlove acquired as arbitrary 8-bit values at per-second mean frequencies of 80.85 ± 0.16 Hz across participants. The average within-participant standard deviation of the sampling frequency was 13.23 Hz. Data shape: *number of samples* × *18*. The column order matches the one from Fig. 1.
- glove_calibrated: Calibrated hand and wrist joint angles in degrees from CyberGlove using the calibration data from “Glove_calibration.xlsx”. Data shape: *number of samples* × *18*. The column order matches the one from Fig. 1.
- fmg: Force myography data from the multimodal bracelet by Andreas *et al*.^38^ acquired as arbitrary 12-bit values at per-second mean frequencies of 89.44 ± 0.02 Hz across participants. The average within-participant standard deviation of the sampling frequency was 3.80 Hz. The signal ranges from 0.0 V to 3.3 V, with higher voltages corresponding to higher forces. Data shape: *number of samples* × *24 (number of FMG modules channels* × *4)*. The order of the FMG channels matches the one from Fig. 4: fmg_1_1, fmg_1_2, fmg_1_3, fmg_1_4, fmg_2_1, fmg_2_2, ...

## Technical Validation

**Fig. 6:**
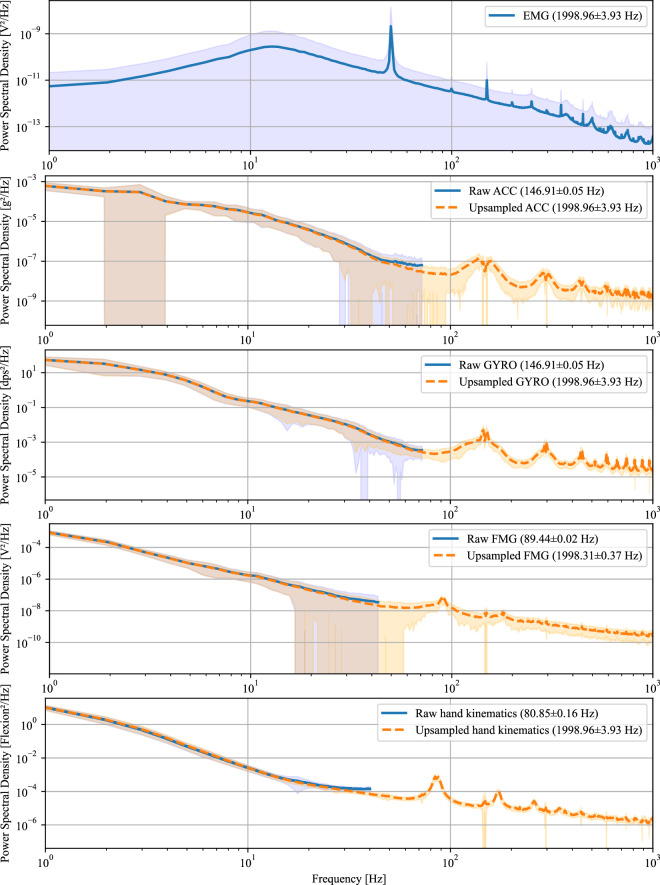
Power spectral density (PSD) comparison between raw data (blue line) and upsampled data using zero-order hold (ZOH) (orange dashed line). PSDs were estimated using Welch’s method and averaged across participants. Standard deviations are marked by the shaded areas.

To assess the effect of temporal alignment after upsampling via zero-order hold, we compared the frequency spectra of all acquired signal modalities before and after alignment. As shown in Fig. 6, the aligned signals retained the key spectral components, confirming that the alignment procedure preserved signal integrity without introducing significant artifacts. The frequency spectra observed for each modality align with previously reported dominant frequency ranges, with EMG signals showing the highest power primarily below 500 Hz and all other modalities (FMG, IMU, and hand kinematics) predominantly below 10-20 Hz^41–44^. Notably, the EMG signal exhibits strong power line interference at 50 Hz and its harmonics, for which the application of a notch filter is recommended.

**Fig. 7:**
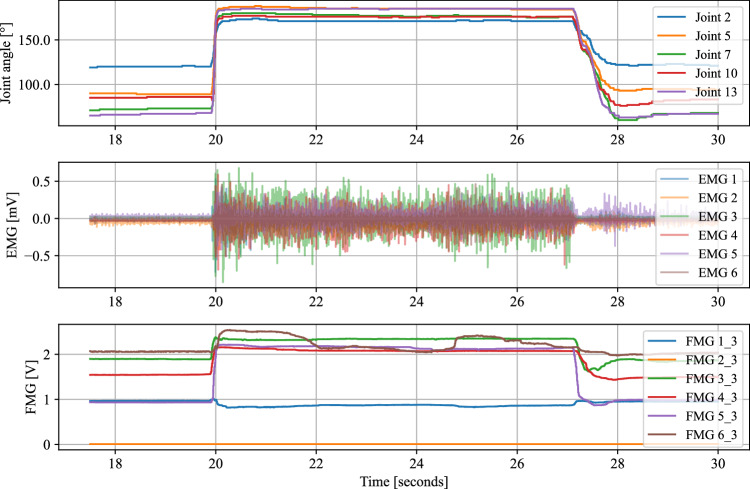
The plot displays the calibrated angles of all MCP joints, alongside EMG data (channels 1-6) and FMG data (channel 3 of each module) over time, captured during a rapid transition from a resting hand position to a fist gesture and back to rest.

To assess the synchronicity of the acquired signals, we instructed one participant to move from a resting hand position to a fist as quickly as possible. After holding the fist position for a few seconds, the participant returned to the resting position again. This rapid muscle activation helps to identify the onset of the motion in the hand kinematics data and thus allows the evaluation of the synchronicity between the EMG and FMG signals. The plot in Fig. 7 from this experiment shows no visible delay between the signals. Notable changes in muscular activity are observed in both the EMG and FMG signals at the onset of the movement, and these changes are well-aligned with the finger motions recorded from all five MCP joints by the CyberGlove.

To quantify the delay between the signals, EMG, FMG, and hand kinematics data by the CyberGlove were low-pass filtered at 10 Hz using zero-phase digital filtering to avoid phase distortion. The EMG signal was additionally bandpass filtered (20-450 Hz, 4th order Butterworth) to remove movement artifacts and high-frequency noise, followed by a rectification. The time window for delay estimation between signals was visually preselected between 19.82 s and 20.08 s (with respect to the time axis from Fig. 7). Delay estimation was then performed using a local slope-sign change-based event detection approach, marking the onset of the action within each signal. Specific channels were selected for the delay estimation based on known anatomical and functional relationships between modalities. Specifically, EMG channel 6, targeting the Flexor digitorum superficialis (FDS), was chosen, along with the four spatially corresponding FMG channels from the same module (see Fig. 4). The FDS is primarily responsible for MCP flexion, corresponding to CyberGlove channels 5, 7, 10, and 13 (see Fig. 1). For FMG and hand kinematics, the signals were averaged across the selected channels. Then, for each signal, the first local minimum within the analysis window was identified, marking the onset of the motion. The delays between modalities were then calculated as the time differences between these detected events. The resulting average delays were: EMG → FMG = 2.22 ms, FMG → Motion = 12.66 ms, and EMG → Motion = 14.89 ms. These values are mostly consistent with physiologically reported benchmarks, including the delay from muscle activation to fascicle motion onset (~6 ms, reflecting the EMG → FMG delay), the delay from fascicle motion to force production onset (~5 ms, corresponding to the FMG → Motion delay), and the overall electromechanical delay from muscle activation to force production onset (~11 ms, reflecting the EMG → Motion delay)^45,46^.

However, it is important to note that these delay estimates should be interpreted with caution. Given the use of surface EMG electrodes and the anatomical placement shown in Fig. 4, cross-talk from overlying muscles such as the Flexor carpi radialis (FCR) and Palmaris longus (PL) may have influenced the EMG recordings and consequently the estimated delays. Although the temporal ordering of the signal onsets appears physiologically correct, the absolute delays slightly differed from those reported in previous studies. Additionally, zero-order hold (ZOH) upsampling may have introduced minor timing inaccuracies due to frequency mismatch between the source and target sampling rates. Although accelerometer (ACC) and gyroscope (GYRO) signals are more difficult to validate for synchronization due to less distinct signal features, their direct recording from the same device as the EMG signals ensured that they remained temporally aligned with the EMG, and thus with the FMG and CyberGlove data. All signals were timestamped using a common time server, making significant timing errors due to clock drift or server inaccuracies unlikely. Overall, these results provide evidence for effective signal synchronization across all modalities used in this study.

To assess the data quality of the MyoKi database objectively, we replicated the acquisition protocol from database 7^11^ of the established Ninapro database (referred to as NinaproDB7 in the following), using the same sensor layout and acquisition setup as shown in Figs. 3, 4, and 5. This approach allows for a direct comparison between the MyoKi database and the established NinaproDB7, providing insights into the reliability of our sensor layout and the quality of the acquired signals. We employed a Long Short-Term Memory (LSTM)^47^ neural network to decode the input signals (EMG, IMU, and FMG, when available) into continuous joint flexion recorded by the CyberGlove. The LSTM network utilized a Rectified Linear Unit (ReLU) activation function, and its hyperparameters were optimized using both the NinaproDB7 and MyoKi databases (number of layers: 2, hidden size: 4096, dropout rate: 0.1, batch size: 128, learning rate: 5.72589e-05). For training, the network was run for 200 epochs with an Adaptive Motion Estimation (Adam) optimizer, a weight decay of 0.0001, and mean squared error (MSE) loss. The input signals were processed with a 250 ms window size and 150 ms overlap. EMG signals underwent preprocessing with a Butterworth filter (low cutoff: 20 Hz, high cutoff: 500 Hz) and a notch filter at 50 Hz to eliminate power line noise. Data from each input channel and hand kinematics data were then normalized to a mean of 0 and a standard deviation of 1. The following features were extracted from their respective input signals to serve as inputs for the LSTM network:

- Electromyography (EMG): Mean Value (MV), Variance (VAR), Root Mean Square (RMS), Signal Range (SR), Waveform Length (WL), Zero Crossing (ZC), Mean Frequency (MNF), Median Frequency (MDF), Spectral Entropy (SE), Skewness (Skew), Kurtosis (Kurt), Entropy, Mean Absolute Value (MAV), Integrated EMG (IEMG), Slope Sign Change (SSC), Log Determinant (LogDet), Difference Absolute Standard Deviation Value (DASDV), and Average Amplitude Change (AAC).
- Accelerometer (ACC): MV, VAR, RMS, SR, WL, ZC, MNF, MDF, SE, Skew, Kurt, Entropy, MAV, IEMG, SSC.
- Gyroscope (GYRO): MV, VAR, RMS, SR, WL, ZC, MNF, MDF, SE, Skew, Kurt, Entropy, MAV.
- Magnetometer (MAG): MV, VAR, RMS, SR, WL, ZC, MNF, MDF, SE, Skew, Kurt, Entropy, MAV.
- Force Myography (FMG): MV, VAR, RMS, SR, WL, ZC, MNF, MDF, SE, Skew, Kurt, Entropy, MAV, IEMG, SSC.

**Fig. 8:**
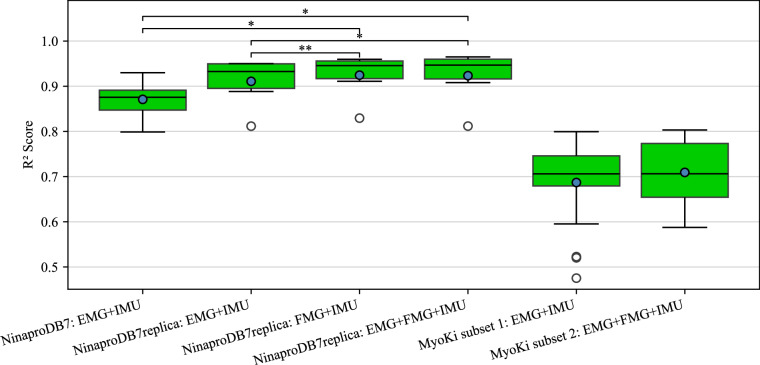
R^2^ scores of the LSTM network decoding input data (combinations of EMG, FMG, and IMU) of NinaproDB7, NinaproDB7replica, and the MyoKi database into 18 continuous wrist and finger joint angles. The blue circles mark the average scores for each database across participants (applies to the NinaproDB7 and the MyoKi database) or across folds when k-fold cross-validation was applied (applies to NinaproDB7replica). The central line in each box represents the median, the lower and upper edges of the box indicate the 25th and 75th percentiles (interquartile range), and the whiskers extend to the most extreme data points not considered outliers (shown as individual points). Significance bars above the boxes indicate statistically significant differences between conditions (*for p < 0.05, **for p < 0.01), as determined by the appropriate statistical test (paired t-test, independent t-test, or Mann-Whitney U test, depending on data structure and normality). Only significant comparisons are shown.

For each participant across all databases (NinaproDB7, NinaproDB7replica, and both MyoKi subsets), the data was split by repetition, with four repetitions used for training, one for validation, and one for testing. For NinaproDB7, the repetitions were split randomly across all 20 participants without amputation. The same was applied for both MyoKi subsets, where repetitions were also split randomly across participants. In contrast, a k-fold cross-validation approach was used for the single participant who replicated the NinaproDB7 acquisition protocol to account for variations across repetitions. In this case, 6 folds were created where each repetition served as a test set once, while the remaining repetitions were randomly divided into training and validation sets. Figure 8 presents the performance of the LSTM network as R^2^ scores (coefficient of determination) across participants or folds in decoding the input data from the test set of each participant (one full repetition of each task including transitions) into continuous hand motions represented by joint flexion. Those values were then compared to the ground truth values recorded by the CyberGlove to compute the R^2^ scores for each participant. The graph presents the performance metrics for NinaproDB7 and its replica (NinaproDB7replica) using our sensor layout, enabling a direct comparison. Additionally, it includes the decoding performance on both subsets of our MyoKi database. It is important to note that the IMU data in NinaproDB7 consists of 3-axis accelerometer, gyroscope, and magnetometer data, whereas both NinaproDB7replica and the MyoKi database contain only 3-axis accelerometer and gyroscope data. Moreover, to ensure a fair comparison, we used uncalibrated hand kinematics data across all databases, as NinaproDB7 does not provide calibrated hand kinematics data.

To compare R^2^ scores between experimental conditions, we first assessed normality using the Shapiro-Wilk test for each pairwise comparison. For comparisons among the three NinaproDB7replica input data combinations, which are based on the same population, we used the paired t-test as all differences were normally distributed. For comparisons between independent groups (NinaproDB7 vs. NinaproDB7replica and MyoKi subsets), we used the independent t-test if both groups were normally distributed, and the Mann-Whitney U test otherwise.

Figure 8 shows higher mean R^2^ scores on NinaproDB7replica for all tested input data combinations (EMG+IMU: 0.911 ± 0.055, FMG+IMU: 0.924 ± 0.050, EMG+FMG+IMU: 0.923 ± 0.058) compared to the original NinaproDB7 (EMG+IMU: 0.871 ± 0.035). However, only the differences of the input combinations of FMG+IMU (Mann-Whitney U, p = 0.011) and EMG+FMG+IMU (Mann-Whitney U, p = 0.019) of NinaproDB7replica were significant. The graph also shows significantly higher R^2^ scores for the input data combinations FMG+IMU (Paired t-test, p = 0.005) and EMG+FMG+IMU (Paired t-test, p = 0.025) compared to EMG+IMU for NinaproDB7replica, highlighting the potential importance of FMG data for robust and reliable hand and wrist motion decoding. These results imply that the signal quality from our data acquisition is at least on par with the established NinaproDB7.

The results further show a notably lower decoding performance on the presented MyoKi database (subset 1: 0.685 ± 0.089, subset 2: 0.709 ± 0.076) compared to NinaproDB7 using the same neural network. This performance difference likely originates from the higher task complexity in our database that involves whole body motions, but also different arm orientations that can lead to electrode shift with respect to the underlying muscles. Besides task complexity, the main difference between the acquisition protocol to acquire NinaproDB7^11^ and our database is the order in which tasks were executed. For NinaproDB7, tasks were each repeated 6 times consecutively before continuing with the next task. In contrast, we asked participants to perform each task once and then repeat the whole taskset 6 times to strike a more even distribution of muscle fatigue across tasks, which is known to impact EMG signals^48,49^.

Looking at the decoding performance differences between both subsets of the MyoKi database shows no significant improvement in using FMG as additional input data along with EMG and IMU (Mann-Whitney U, p = 0.571). However, a direct comparison is only possible when comparing different input signals on the same set of participants. With the added subset that includes FMG data, the MyoKi database gives researchers the tools to investigate the benefits of different input signals to control robotic and prosthetic hands in the future.

## Usage Notes

The primary goal of this database is to support research on improving continuous control of individual joints in robotic, virtual, or prosthetic hands using muscular activity. In regression-based approaches, neural networks are commonly used to decode the muscular activity from a user’s arm into joint movements. To achieve this, EMG, FMG, and IMU data serve as input signals, which are then mapped to finger and wrist joint angles recorded by the CyberGlove (used as ground truth). The precise labeling allows the use of the database not only for regression but also for classification by mapping each task to the used grasp according to the table in “Task_categorization.xlsx”. It is important to note that the EMG signals in this database are not pre-filtered. A 50 Hz notch filter is typically applied to remove power line interference, along with a bandpass filter (20-500 Hz) to enhance the signal-to-noise ratio.

The MyoKi database offers two options for hand kinematics data: non-calibrated data with 8-bit arbitrary values representing joint flexion or calibrated data with joint angles in degrees [^°^]. Since neural networks are robust to linear scaling, choosing between calibrated and uncalibrated data should not affect decoding performance. The calibrated joint angles by the CyberGlove, however, can be useful for kinematic analysis or reconstructions of hand motions during daily tasks. Since generalized calibration data was used instead of participant-specific calibration, there is a potential for inaccuracies arising from differences in hand shape and size among participants. However, a previous study showed that generalized calibration data can perform sufficiently well^36^. The kinematics data, covering 18 hand and wrist joints, can further offer insights into which joints or tasks are more challenging to decode and whether performance is influenced by task complexity, required force, or motion distance.

The database also includes demographic and anthropometric information of each participant, enabling analysis of how factors such as sex, age, and body fat influence decoding performance. Moreover, by providing exact sensor locations it allows researchers to examine the role of specific muscle regions in hand and wrist control. The MyoKi database comprises two subsets (subset 1: P01 to P25, subset 2: P26 to P35), which share an identical data acquisition protocol. The only difference is that for subset 2, additional FMG data was recorded. The multimodal myography data facilitates comparisons between different input signal modalities, helping to determine their relative importance for motion decoding.

## Data Availability

To acquire FMG data for subset 2 of the MyoKi database, the updated version (v2) of the multimodal bracelet by Andreas *et al*.^38^ was used. The repository (https://github.com/ASM-FAU/Multimodal-Bracelet) contains CAD models to 3D print the bracelet, a circuit diagram for the PCB, and provides code to collect data and save it at a host PC wirelessly or through a USB serial connection. Further, this repository also contains the CAD models of objects used in the experimental setup, such as the block with a vertical and horizontal screw or the 3D-printed smartphone with the target layout to simulate tapping. The typing test used in this work is from https://10fastfingers.com/, and the mouse click test can be downloaded from http://www.yorku.ca/mack/FittsLawSoftware/(used settings for a 24-inch 1080p monitor: FittsTask 2D with an amplitude of 750 pixels, and a target width of 30 pixels).
